# Computer-assisted correction of incongruent distal radioulnar joints in patients with symptomatic ulnar-minus variance

**DOI:** 10.1177/17531934221091870

**Published:** 2022-06-14

**Authors:** Lea Estermann, Lukas Urbanschitz, Lisa Reissner, Andreas Schweizer

**Affiliations:** Department of Orthopedics, Balgrist University Hospital, Zurich, Switzerland

**Keywords:** Ulnar-minus variance, ulnar negative variance, radius shortening osteotomy, ulnar lengthening osteotomy, DRUJ reconstruction

## Abstract

Our study described a computer-assisted, three-dimensional (3-D), planned surgical technique of a radial shortening osteotomy. The osteotomy of the distal radius was planned with computer assistance on 3-D bone models based on computed tomography data. The objective was to maximize the contact zone of the sigmoid notch with the ulnar head. Between 2012 and 2020 we treated 14 wrists in 11 patients with symptomatic ulnar-minus variance with a mean follow-up of 44 months (range 8 to 98) and a mean age of 28 years (range 19 to 38). Postoperatively, patients showed a decrease in pain at rest and during effort (numeric rating scale from 4.4 to 0 and 7.5 to 4.5, respectively). The range of motion postoperatively was similar to the contralateral side. Grip strength increased from 24 kg to 30 kg. The Disability of the Arm, Shoulder, and Hand and the Patient-Rated Wrist Evaluation scores were 28 and 35 postoperatively, respectively. Our technique of 3-D computer-assisted distal radioulnar joint reconstruction led to a pain reduction and improvement of the hand function in patients with symptomatic ulnar-minus variance.

**Level of evidence:** IV

## Introduction

Joint levelling procedures aim to decrease ulnar-sided wrist pain, especially in patients with a considerable ulnar-plus variance. There is a wide range of ulnar variance in adults, ranging from −4.2 mm to +2.3 mm with a mean ulnar variance of −0.9 mm (Schuind et al., 1992). It is unclear how much variance is tolerated before symptoms occur (Cannata et al., 2003; Golz et al., 1991). Available evidence on how to address a severe symptomatic ulnar-minus variance is sparse. Ulna distraction osteogenesis and ulnar lengthening osteotomy, especially corrections with external fixation, have been described mostly in young patients with hereditary multiple exostoses or after growth arrest due to a fracture or infection of the distal ulnar physis (Abe et al., 1996; Bader and Grill, 2000; Cho and Jung, 2014; D’Ambrosi et al., 2016; Mader et al., 2003; Müller-Färber and Schläger, 2008; Pritchett, 1986; Tang et al., 2013; Waters et al., 1997). In addition, there were two published cases of mild ulnar longitudinal deficiency that were successfully treated by an ulnar z-shaped lengthening osteotomy (Farr and Schachinger, 2020).

Radial shortening osteotomies are considered as joint levelling procedures and radial decompression osteotomies as de-tensioning procedures of the distal radioulnar joint (DRUJ) through an ulnar translation of the radius shaft (Krimmer, 2018; Krimmer et al., 2016; Müller-Färber and Schläger, 2008), mainly described for treatment of early Kienböck’s disease. Nonetheless, there is little evidence for management of severe ulnar-minus variance (Koh et al., 2003; Matsui et al., 2014; Watanabe et al., 2008). Although the technique for radial decompression osteotomies has been described, no treatment results were reported (Krimmer, 2018; Krimmer et al., 2016).

Recent developments in computer-assisted, three-dimensional (3-D) planning and printing have led to the use of customized instruments for individual patients, which help to accurately correct even complex malunions of the forearm (Bauer et al., 2017; Vlachopoulos et al., 2015). The computer-assisted planning of the osteotomy enables a correction of the dorsovolar tilt, radial inclination, rotation and translation. The contact zone in the sigmoid notch can be evaluated and adjusted three-dimensionally to achieve the best match with the ulna head. We hypothesized that the re-establishment of this contact zone leads to an improved congruency of the DRUJ and thereby reduces pain and improves hand function. The primary objective of this study was to describe our protocol for 3-D planned reconstruction of the DRUJ to improve congruency through radius shortening with or without ulnar lengthening procedures. We also report the results of postoperative pain, ulnar variance, range of motion (ROM), grip strength and patient-reported outcomes using this protocol.

## Methods

In this retrospective study, we searched the clinic’s database for all patients who underwent 3-D planned realignment surgery of the DRUJ for constitutional or post-traumatic ulnar-minus variance between August 2012 and March 2020. Baseline data and those from the last follow-up for ROM and grip strength were extracted from patient records by an independent hand surgeon (expertise Level 2) (Tang and Giddins, 2016). The same hand surgeon assessed pain using the numeric rating scale (NRS) from 0 (no pain) to 10 (worst pain) and the Single Assessment Numeric Evaluation (SANE) scores (Gire et al., 2020) via telephone interviews. The patient-reported outcome was analysed with the Patient-Rated Wrist Evaluation (PRWE) and the Disabilities of the Arm, Shoulder, and Hand (DASH) score via postal questionnaires.

Pre- and postoperative ulnar variance values were measured on digital anterior–posterior radiographs by means of the method of perpendiculars as described by Coleman et al. (1987) by the same hand surgeon who performed the telephone inquiry. The amount of angular correction and shortening of the radius was extracted from the preoperative 3-D-planning as described below. This study was approved by the local ethics committee, and all patients provided written informed consent.

### Preoperative planning and surgical technique of radius shortening and ulna lengthening osteotomies

**Figure 1.**
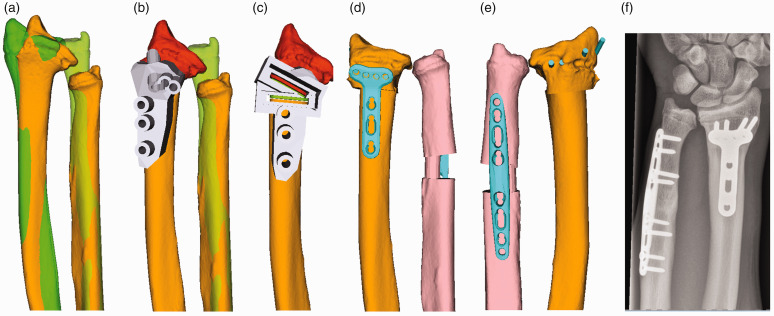
Post-traumatic severe ulnar-minus variance with preoperative computer-assisted, three-dimensional planning of the radius osteotomy, distal radioulnar joint (DRUJ) reconstruction and ulnar lengthening. (a) Three-dimensional bone model of pathologic DRUJ (orange), uninjured contralateral side (green), (b) drill guide (grey) and distal part of the radius (red), (c) saw guide to remove a bone wedge, (d) anterior view of the reconstructed DRUJ with distal radius plate and long ulnar shortening plate (mint), (e) dorsal view of the reconstructed DRUJ, (f) postoperative anterior–posterior radiographs of the wrist.

**Figure 2.**
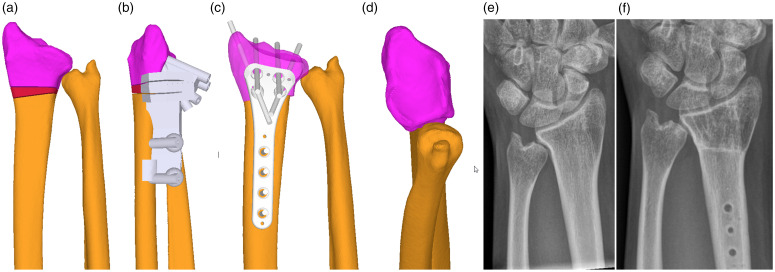
Congenital ulnar-minus variance with preoperative computer-assisted, three-dimensional planning of the radius osteotomy and distal radioulnar joint (DRUJ) reconstruction. (a) Three-dimensional bone model of pathologic DRUJ, proximal radius and ulna (orange), bone wedge (red), distal radius (pink), (b) drill and saw guide (grey), (c) distal radius plate (grey), (d) articular view of the reconstructed DRUJ, (e) preoperative anterior–posterior radiographs of the wrist, (f) postoperative anterior–posterior radiographs of the wrist.

Bilateral computer tomography (CT) scan images were segmentally obtained using commercially available software (Mimics, Materialise, Leuven, Belgium). Based on these, 3-D bone models of both forearms and wrists were created. The osteotomy of the distal radius was planned with computer assistance based on the contralateral radius or a standard model in cases with bilateral DRUJ incongruences. Correction of the angulation (rotation, radioulnar and dorsovolar tilt) as well as translation and shortening were simulated and adjusted manually to increase the joint contact zone. Afterwards, design and production of cutting guides to remove a bone wedge and drilling guides (Medacta SA, Castel San Pietro, Switzerland) for precise screw holes for the future plate were performed (Figures 1 and 2). In cases with higher ulnar-minus variances, a concomitant ulnar lengthening was performed to minimize the length differences of the forearms. The osteotomies were carried out by one senior hand surgeon with Level 4 expertise (Tang and Giddins, 2016).

The radius was exposed through a standard modified Henry approach. The surface of the radius was carefully prepared for a precise fit of the guides. The shortening osteotomy of the radius was performed and fixed with anterior radius plates. The plates used included Correctus (five), Primus (one) (Intercus AG, Aarau, Switzerland)), distal radius (four) (Synthes GmbH, Obersdorf, Switzerland), distal radius (two) (Medartis®, Basel, Switzerland) and a locking 2.7 mm compression plate (one) (Synthes GmbH, Obersdorf, Switzerland). In two patients, an ulnar lengthening was performed using an approach between the flexor carpi ulnaris and extensor carpi ulnaris muscle. The same technique as described above with drill and saw guides was used, and a long ulnar shortening plate (Synthes GmbH, Obersdorf, Switzerland) was used. Radius bone graft of the shortening osteotomy (in two patients) and additional cancellous bone graft from the iliac crest (one patient) was used to fill the ulnar defect. After fluoroscopy verification of congruency of the DRUJ, the wound was closed, and an anterior splint applied. After 2 weeks, unloaded mobilization was started under the guidance of a hand therapist. Eight weeks postoperatively, a CT scan was performed to check for consolidation.

### Statistical analysis

All data were tested with the Kolmogorov–Smirnov-test for normal distribution. Normally distributed data are presented as mean and standard deviation (SD); non-normally distributed data as median, interquartile range (IQR) and range. Due to the small patient numbers, the comparison between operated and contralateral sides was carried out with Wilcoxon-test. The level of significance was set to *p* ≤ 0.05. A post-hoc power analysis was conducted.

## Results

A total of 14 radial shortening and adjusting osteotomies for symptomatic DRUJ incongruencies were performed using the above-described protocol. Preoperatively, patients complained about localized pain at the DRUJ during pronation and supination and when lifting heavier objects. Two patients could not be reached by phone but filled in the postal questionnaires; therefore, pain and SANE score were evaluated for nine patients (12 forearms). The mean postoperative follow-up for the questionnaires by telephone and mail was 44 months (SD 28; range 8 to 98), and for the ROM and radiologic assessment was 20 months (SD 18; range 6 to 61) and 20 months (SD 17; range 2 to 56), respectively. Three patients were men and eight were women with a mean age of 28 years (SD 7; range 19 to 38) at follow-up. Four extremities had post-traumatic deformities, and ten had ulna dysplasia (Table 1). All forearms underwent radius shortening osteotomy, and two had an additional ulna lengthening due to insufficient realignment with a single osteotomy (Figure 1 and 2). None of the patients had peri- or postoperative complications; in 12 forearms, however, the implants were removed because of soft tissue irritation due to the hardware and/or the young age of the patients. The median time until consolidation was 9 weeks (IQR 8 to 12; range 7 to 18).

**Table 1.** Pathology, pre- and postoperative ulnar variance, and the computer-assisted planned angular correction in three dimensions.

Case	Pathology	Preoperative UV (mm)	Postoperative UV (mm)	Angular correction: pronation (°)	Angular correction: extension (°)	Angular correction: varus (°)	Longitudinal radius correction (mm)	Additional ulnar lengthening (mm)
1	Post-traumatic	–11	–5	–3	–4	–6	–8	
2	Ulna dysplasia	–5	–1	0	–0	–6	–6	
3	Ulna dysplasia	–7	0	–5	–2	–4	–9	
4	Ulna dysplasia	–2	–1	0	0	0	–2	
5	Ulna dysplasia	–5	0	0	–1	–6	–5	
6	Ulna dysplasia and post- traumatic	–5	–1	11	–7	1	–3	
7	Post-traumatic	–12	0	13	1	–10	–7	9.8
8	Ulna dysplasia	–3	1	–0	3	1.0	–3	
9	Ulna dysplasia	–6	–1	9	5	11	–3	
10	Post-traumatic	–7	0	–20	12	–22	–3	11.8
11	Ulna dysplasia	–9	0	0	–2	–5	–8	
12	Ulna dysplasia	–8	0	0	0	5	–8	
13	Ulna dysplasia	–4	–2	–5	–1	–8	–4	
14	Post-traumatic	–6	0	–11	7	–5	–4	
	Median (IQR)	–6 (–8 to –5)	0 (–1 to 0)	0 (–5 to 3)	–0 (–2 to 4)	–5 (–6 to 1)	–5 (–8 to –3)	10.8

UV: ulnar variance, IQR: interquartile range.

Table 1 shows the pre- and postoperative ulnar variance and planned corrective angles. In case number 10, the planned amount of shortening of the radius and lengthening of the ulna was overestimated, so the ulnar lengthening was reduced intraoperatively. The reason for the overestimation may be the complexity of 3-D planning of a simultaneous radius shortening and ulnar lengthening osteotomy, and the difference between the 3-D preoperative planning and 2-D intraoperative imaging for ulnar variance measures.

Table 2 shows the comparison of pain at rest and during effort, showing a significant decrease of both parameters postoperatively. In those patients with a unilateral osteotomy, when comparing the postoperative ROM with the normal side, there was a significantly lower degree of flexion in the operated extremities (Table 3). Postoperative grip strength of the surgically treated side (mean 30 kg; SD 11 kg) when compared with the contralateral asymptomatic side (mean 35; SD 9 kg; *p* = 0.05) was significantly lower. However, when comparing preoperative (mean 24 kg; SD 9 kg) and postoperative values (mean 30 kg; SD 11 kg), grip strength increased significantly (*p* = 0.016).

**Table 2.** Pain and patient-reported outcome.

	Preoperative	Postoperative	*p*-value
Median pain at rest (NRS)	4 (IQR, 3 to 7.5)	0 (IQR, 0 to 2)	0.003
Median pain during effort (NRS)	7.5 (IQR, 7 to 10)	4.5 (IQR, 2 to 5)	0.006
Mean SANE score	49% (SD, 19)	77% (SD, 15)	0.008
Mean DASH		28 (SD, 22)	
Mean PRWE		35 (SD, 22)	

IQR: Interquartile range; NRS: numeric rating scale; SANE: Single Assessment Numeric Evaluation; DASH: Disabilities of Arm, Shoulder and Hand; PRWE: Patient-Rated Wrist Evaluation.

**Table 3.** Comparison of range of motion of treated and contralateral asymptomatic side.

	Surgically treated Median (IQR)	Control Median (IQR)	*p*-value
Flexion (°)	75 (70 to 85)	80 (70 to 90)	0.038
Extension (°)	80 (60 to 80)	70 (68 to 80)	0.395
Pronation (°)	80 (68 to 80)	80 (70 to 80)	0.713
Supination (°)	80 (80 to 90)	80 (78 to 90)	0.194
Ulnar deviation (°)	40 (30 to 43)	35 (33 to 43)	1
Radial deviation (°)	20 (10 to 28)	20 (15 to 23)	1

Regarding functional postoperative outcome, the mean DASH score was 28 (SD 22) and the mean PRWE was 35 (SD 22). The SANE score showed a significant increase postoperative (Table 2). All patients with the exception of one, would repeat the surgery according to the given outcome. In this one patient who had undergone bilateral radius osteotomies, she expressed satisfaction with the right hand and would repeat the surgery, but remained dissatisfied with the left side because of persistent pain during pronation and supination.

## Discussion

This study demonstrated that a computer-assisted, 3-D-planned DRUJ reconstruction with radial shortening osteotomy with or without an ulnar lengthening procedure can reduce pain and improve hand function in patients with symptomatic ulnar-minus variance. Our investigation of 14 cases with ulnar-minus variances between –12 and –2 mm showed that this deformity is not always well tolerated without treatment, in contrast to the study by Cannata et al. (2003). Our findings of pain reduction through a joint levelling/adjusting procedure are in line with previous findings (Bader and Grill, 2000; D’Ambrosi et al., 2016). Ulnar-minus variance with DRUJ incongruency might lead to ulnar-sided wrist pain due to an impingement of the ulnar head with a non-articular surface of the distal radius and an overload because of an altered force transmission across the wrist.

D’Ambrosi et al. (2016) and Tang et al. (2013) reported an improvement of pronation and supination postoperatively after ulnar lengthening procedures. Most of our patients did not show a significant impairment of the ROM preoperatively and therefore no significant postoperative improvement was found. Although we found a significant postoperative improvement in grip strength, we were unable to compare our results with other studies as, to our knowledge, there is no published data available for comparison.

The improved SANE score from 49% to 77% shows that the function of the wrist can be improved but not normalized through a DRUJ reconstruction. The lack of normalization but functionality of the wrist is also reflected in the PRWE and DASH score of 35 and 28, respectively.

Our technique aims to restore normal anatomy, which increases the tension of the intraosseous membrane and consequently prevents DRUJ instability. Conversely, this might lead to an overpressure at the DRUJ. In contrast, this risk does not exist using the technique from Krimmer et al. (2016) who describe the DRUJ de-tensioning procedure with radius osteotomy and ulnar translation and shortening of the radius shaft resulting in loosening of the distal oblique bundle. However, their technique increases the risk of DRUJ instability and non-anatomical kinematics.

The main limitation of this study is the small number of patients and the varying amount of ulnar variance. Furthermore, two patients could not be reached by phone, so that NRS assessment and SANE score were evaluated from only nine patients. However, due to the large differences between the pre- and postoperative SANE score, the post-hoc power analysis revealed a power of 98% with an alpha error probability of 0.05. Changes in the NRS yielded a power of 100% during rest and 99% during effort, respectively. Preoperative patient-reported outcome scores were not available; therefore, a complete preoperative versus postoperative comparison could not be made.

Our study showed the efficacy of 3-D-assisted planning in the management of incongruent DRUJs in patients with symptomatic ulnar-minus variance by DRUJ reconstruction resulting in pain reduction at rest and during effort and improvement of mobility and function of the hand.
